# Is the Victimization-Perpetration Association for School Bullying a Cycle of Bias?

**DOI:** 10.1177/08862605251315778

**Published:** 2025-01-28

**Authors:** Allison Kurpiel

**Affiliations:** 1 The University of Edinburgh, UK

**Keywords:** bias, victimization, school bullying, perpetrator, victim-offender, prejudicial

## Abstract

It is well known that some youth are both victims and perpetrators of bullying. However, it remains unclear whether the victim-perpetrator overlap contains specific characteristics, such as bias. Using data from the United States Health Behavior among School-aged Children survey from 2009 to 2010 (*N* = 8,739), this study investigated the victim-perpetrator overlap for school bullying, with emphasis on assessing whether the perpetrators of biased (i.e., bias-motivated or prejudicial) bullying are also victims of biased bullying. The analyses employed predictive modeling using cross-sectional data and multinomial logistic regression to examine whether perpetrating biased bullying is associated with a higher risk of experiencing biased victimization than nonbiased victimization (and no victimization). It was then determined among which demographic subgroup of students, the biased bullying victim-perpetrator overlap is most prevalent. Results indicated evidence of a type-specific victim-perpetrator overlap for biased bullying. The biased bullying victim-perpetrator overlap was most prevalent among females, students whose families have financial difficulties, and students not born in the United States. These findings suggest that bullying perpetrators are not only at risk of being victims of bullying generally, but they are specifically more likely to be victims of the type of bullying they perpetrate. School programming to combat biased bullying should be designed with the understanding that there are some students in both the victim and perpetrator roles. Initiatives should focus on potential avenues for breaking the cycle of bias, especially among the group of students most likely to be involved.

School bullying constitutes a public health concern for young people. Bullying affects approximately 20% of adolescents in the United States (Wang et al., 2020) and can cause substantial harm to victims’ health, well-being, development, and performance in school (Schoeler et al., 2018). One form of school bullying that can have particularly severe consequences is biased bullying. Biased (also called bias-motivated, prejudicial, or stigma-based) bullying occurs when a perpetrator chooses their target based in whole or in part on the target’s actual or perceived characteristics (e.g., sexual orientation, race/ethnicity). Biased bullying can be any form of bullying (e.g., verbal, physical) so long as the victim’s identity is perceived to have motivated the incident. Nonbiased bullying, on the other hand, is any form of bullying where identity is perceived to be unrelated.

Biased victimization is associated with greater negative impacts (e.g., on physical health, self-esteem) for students than nonbiased victimization (Kurpiel, 2024). It can also cause vicarious harm to students who share the victim’s characteristics (Perry & Alvi, 2012) and damage a school climate of inclusivity (Perry, 2015). As institutions of socialization, schools are important avenues by which prejudice and stereotypes are prevented or perpetuated among the next generation (Peguero, 2012). Hate-motivated, intergroup conflict has given rise to some of history’s worst atrocities (e.g., genocide), so it is important to intervene when the prejudice is still forming during childhood.

Research has established in that some youth involved in bullying are “victim-offenders,” in that they both bully others and are bullied themselves (i.e., “bully-victims”; Menesini & Salmivalli, 2017). Most work on bully-victims has focused on the health and behavioral outcomes associated with being both a victim and perpetrator of bullying (relative to being a victim only or perpetrator only; e.g., Silva et al., 2013). Only a small number of studies (e.g., Stubbs-Richardson & May, 2021) have examined whether particular types of bullying perpetration are associated with victimization by that same type of bullying, or *specificity* in the bullying victim-offender overlap. The focus of the current study is on a potential type-specific victim-perpetrator overlap for biased bullying.

It is important to understand whether some youth are both victimized by and perpetrators of biased bullying because such a finding would have both theoretical applications regarding the etiology of biased bullying and practical implications regarding how schools should target their limited resources toward reducing this harmful behavior. Knowledge of whether biased bullying is experienced by those who perpetrate it is crucial for understanding the nature of prejudiced acts in the school setting.

Very little prior work on biased bullying has examined a potential victim-perpetrator overlap, and studies that have either focused on one bias type or used nonrepresentative samples (e.g., Galán et al., 2021; Hatchel et al., 2020). The paucity of research on this topic is especially concerning in the U.S. context given the relatively high rate of hate crimes committed in the United States (Levin & Reitzel, 2018), which biased bullying could escalate to if unaddressed (Englander, 2007). To fill this gap, the current study used data from the United States Health Behavior among School-aged Children survey from 2009 to 2010 (*N* = 8,739) to examine the association between biased bullying perpetration and biased bullying victimization. It was then determined among which subgroups of the student population the biased bullying overlap is most prevalent.

## Understanding Biased Bullying Perpetration and Victimization

### Biased Bullying Perpetration

A leading explanation for biased aggression among youth points to thrill-seeking as the primary goal (Levin & McDevitt, 1993). Thrill-seekers usually choose victims who present easily identifiable, stigmatized social characteristics. Though both general and biased peer victimization are often perpetrated to gain power or status at the expense of their victim (Faris & Felmlee, 2014), the thrill from perpetrating *biased*—as opposed to nonbiased—bullying arises from the offender’s display of normativity to peers (Levin & Reichelmann, 2015). Since biased incidents involve the demeaning of socially devalued identities, perpetrating biased bullying reinforces one’s own identity as superior (Perry, 2015).

The approval of peers is a major concern for young people. It may, therefore, be unsurprising that students are susceptible to perpetrating biased bullying to elevate their status (Stotzer, 2015). Indeed, Ballaschk et al. (2021) found that feelings of inferiority as well as fear of diminishing status were commonly reported reasons for hate speech perpetrated by youth in Germany. Prior literature also indicates that being male and an immigrant are associated with the perpetration of bullying based on ethnicity (Bayram Özdemir et al., 2020).

### Biased Bullying Victimization

To understand the risk of biased victimization, researchers draw on routine activities theory (Ellonen et al., 2021). Routine activities theory posits that an individual’s risk of victimization is determined by the amount of time they spend in situations lacking capable guardianship where they are a suitable target in the presence of a motivated offender (Cohen & Felson, 1979). Target suitability refers to the extent to which an individual is perceived to be vulnerable to victimization (as indicated by factors such as physical size).

Expanding on the concept of target suitability, Finkelhor and Asdigian (1996) offered the notion of “target congruence” to help researchers identify risk factors for specific forms of victimization. According to target congruence, personal characteristics can increase an individual’s target suitability by matching up with an offender’s motivations. For biased victimization, socially devalued characteristics (e.g., non-White ethnicity) might increase the risk of biased victimization in the presence of an offender motivated by prejudice (Ellonen et al., 2021). Evidence also suggests that youth born outside of the United States (Maynard et al., 2016), females (Price-Feeney et al., 2018), and overweight youth (Bucchianeri et al., 2013) have a heightened risk of being victimized by biased bullying.

## The Victim-Offender Overlap and Biased Bullying

### The Victim-Offender Overlap

Explanations for the victim-offender overlap either point to the causal role of victimization in increasing risk of later offending or emphasize that victimization and offending are explained by the same factors (Pridemore & Berg, 2017). Regarding the former, victimization can lead to negative emotionality, which may translate into lifestyle changes that elevate the risk of offending (Ruback et al., 2014). Alternatively, youth who engage in lifestyles that contain high opportunity for bullying and victimization—lifestyles with low guardianship and high exposure to delinquent peers—have a high risk of victimization as well as offending.

### Potential Explanations for a Biased Bullying Victim-Offender Overlap

While the above frameworks imply a general victimization-perpetration association, such that the perpetration of bullying is associated with increased risk of any form of victimization, other influential frameworks imply that the overlap may be type specific. Social learning theory (Sutherland, 1947) suggests that individuals engage in aggressive behaviors because their interactions with others expose them to antisocial norms that condone those behaviors in that context. Adolescents might also imitate behaviors that they have experienced as victims, as such experiences can result in youth acquiring beliefs favorable to those behaviors (Widom, 1989). As biased bullying is based on *perceived* identities, students may attempt to conceal their identities when perpetrating against others.

Situational opportunity theory suggests that the instantaneous social rewards for certain behaviors are boosted within peer groups (i.e., not through value transference; Weerman et al., 2018). Aggressive behaviors are promoted within a social context if group norms assign status to perpetrators, even when some or most youth do not individually subscribe to those values. Many researchers (e.g., Kärnä et al., 2011) claim that bullying is a group rather than an individual level phenomenon, and bystanders play an important role in encouraging (or not discouraging) bullying (Salmivalli, 2010).

Cultural accounts of the overlap posit that norms about how to react to threats imply the need to defend oneself with aggressive behavior (Berg & Schreck, 2022). It is possible some peer groups contain norms requiring that biased victimization should be reacted to with similar discriminatory attacks. Thus, biased victims may become perpetrators in direct retaliation or for revenge (Frey et al., 2015). In summary, these perspectives suggest that social settings contain norms for aggressive behaviors, and these norms increase the risk of both victimization and perpetration of those behaviors for youth in those settings.

### Previous Research on Type Specificity in the Victim-Offender Overlap

Although most prior work on the bullying victim-offender overlap has focused on a general overlap, a few studies have found that perpetration of a particular type of bullying is positively associated with victimization by the same type of bullying. For example, Stubbs-Richardson and May (2021) found that for verbal, physical, and relational bullying, youth who had perpetrated a type of bullying were more likely to experience that same form of bullying as a victim. Hatchel et al. (2020) found that some youth in their sample who perpetrated homophobic bullying were also targets of homophobic bullying. Another study indicated that biased perpetration is associated with biased victimization among high school students in Pennsylvania (Galán et al., 2021).

## The Current Study

The goal of the present study was to improve understanding of both biased bullying and the victim-offender overlap by examining a potential biased bullying victim-offender overlap in a national sample of youth. Another aim was to determine the demographic of students who are most often involved. The analyses were guided by two research questions:

Research question 1: Is there a positive association between biased perpetration and biased victimization which is greater than the associations between biased and nonbiased victimization and perpetration?
Research question 2: Among which demographic group of school-aged children is the biased bullying victim-perpetrator overlap most prevalent?

Regarding research question 1, perpetration was expected to be positively associated with victimization regardless of the type of bullying, following the bullying literature (Menesini & Salmivalli, 2017). However, if there is type specificity in the victim-perpetrator overlap, such that the overlap is characterized by bias, the association between biased perpetration and biased victimization should be greater than the associations between (1) biased perpetration and nonbiased victimization and (2) nonbiased perpetration and biased victimization. With respect to this expectation of type specificity, the following three hypotheses were tested:

Hypothesis 1: Biased (vs. no) perpetration will be positively associated with biased (vs. no) victimization, and this association will be greater than the association between nonbiased (vs. no) perpetration and biased (vs. no) victimization.
Hypothesis 2: Biased (vs. no) perpetration will be positively associated with nonbiased (vs. no) victimization, but this association will be lesser than the association between biased (vs. no) perpetration and biased (vs. no) victimization.
Hypothesis 3: Biased (vs. no) perpetration will be positively associated with biased (vs. nonbiased) victimization, and this association will be greater than the association between nonbiased (vs. no) perpetration and biased (vs. nonbiased) victimization.

Since previous studies have not examined the characteristics of victim-perpetrators of biased bullying, the analyses pertaining to research question 2 were largely exploratory. However, as some literature indicates that immigrant youth have a higher risk than nonimmigrant youth of both victimization and perpetration of biased bullying, the following was hypothesized:

Hypothesis 4: The biased bullying victim-perpetrator overlap will be most prevalent among youth who were not born in the United States (immigrant youth).

## Method

### Sample

The sample for the current study came from the United States Health Behavior in School-Aged Children (HBSC) survey (Iannotti, 2013; available at https://hbsc.org). The HBSC is a cross-sectional, cross-national study sponsored by the World Health Organization. Each HBSC is nationally representative and collected through a three-stage stratified design. Census divisions and grades are strata and school districts are the primary sampling units. The 2009/2010 U.S. survey was administered to youth in 314 public and private secondary schools (grades 5–10). The 2009/2010 HBSC was analyzed because it contains measures of biased victimization as well as perpetration (other versions contain only victimization). Students in grades 5 and 6 were excluded because they were not asked indicators that may be risk factors for biased bullying due to their young age. The final analytic sample was *N* = 8,739.

Just under half (48%) of students were female (see Table 1). Regarding age, 60% were between 14 and 18 and 40% were ages 10 to 17. The majority of students were non-Hispanic White (hereafter White, 54%), followed by Non-Hispanic Black (hereafter Black, 15%), Hispanic (14%), two or more ethnicities (7%), Asian (4%), and another race/ethnicity (6%). Eight percent were not born in the United States. Family financial hardship (measured by asking the students to report how often they go to bed or school hungry because there is not enough food at home) was experienced (always or sometimes) by 25% of the sample. Most students lived in suburban areas (43%). Twenty-five percent lived in urban and 32% lived in rural areas.

**Table 1.** Weighted, Descriptive Statistics for Analytic Sample, HBSC 2009–2010 (*N* = 8,739).

Variable	Percent/Mean (SE)
Bullying victimization (past couple of months)	
No victimization	49
Any biased	27
Nonbiased only	24
Bullying perpetration (past couple of months)	
No perpetration	60
Any biased	13
Nonbiased only	27
Covariates	
Female	48
Male	52
Age: 10–13	40
Age: 14–18	60
Non-Hispanic White	54
Non-Hispanic Black	15
Hispanic	14
Two or more race/ethnicities	7
Asian	4
Another race/ethnicity	6
Not born in the United States	8
Go to bed/school hungry	25
Urban	25
Suburban	43
Rural/other	32
I am under or over weight	51
Low parental monitoring	18
Nights with friends (per week)	2.25 (0.48)
Been in a fight (past year)	33
Been drunk (past 30 days)	15
Carried a weapon (past 30 days)	15
Any friends drink alcohol	54
Do not like school (1–4)	2.08 (0.02)

*Note.* Estimates weighted to adjust for the complex survey design; 20 imputed datasets. HBSC = Health Behavior in School-aged Children; SE = standard error.

### Procedure

The U.S. HBSC (Iannotti, 2013) was collected by the CDM Group, Inc., Bethesda, MD, through on-site (i.e., in school) questionnaires (roughly 60% online and 40% paper surveys). The 45-min anonymous survey was administered in a classroom by a school representative (e.g., teacher, guidance counselor), who read scripts that explained the survey procedures. Consent was obtained from 98% of students (opt-out), of whom about 90% competed the questionnaire. Use of these data for this study was exempt from institutional review board approval because it is a public, deidentified dataset. However, each country that administers the survey must comply with World Health Organization ethical practice guidelines (see Currie et al., 2010).

### Measures: HBSC Survey

#### Bullying Victimization

An 11-item measure was used to capture bullying victimization. The HBSC survey (Iannotti, 2013) asked the students to indicate how often they have been bullied at school in the past couple of months using 11 items referring to specific bullying behaviors. Eight items referred to bullying behaviors that do not involve bias (e.g., I was left out of things on purpose; I was hit, kicked, pushed, shoved around, or locked indoors; alpha = .83). Three items referred to bullying behaviors that involve bias (e.g., I was bullied with mean names and comments about my race or color; alpha = .65).

To each of the bullying items, the student can respond “I have not been bullied in this way in the past couple of months,” “only once or twice,” “2 or 3 times,” “about once a week,” or “several times a week.” Each bullying measure was dichotomized to equal 1 (yes) if the student reported experiencing that type at least once. The types of bullying were dichotomized because this research focused on whether students were victimized by the same type of bullying they perpetrate, rather than the frequency by which these experiences occur.

Youth were categorized (mutually exclusive) as having experienced no victimization (i.e., not a victim of any type of bullying), biased bullying victimization (i.e., experienced *any* bullying victimization involving bias, which included mean names and comments about race or color, about religion, or sexual jokes, comments, or gestures), or nonbiased bullying victimization (i.e., experienced only bullying that did not involve bias).

#### Bullying Perpetration

Bullying perpetration was measured in the HBSC survey (Iannotti, 2013) identically to how bullying victimization was measured, except the question asked whether the student bullied other students in the specified ways (rather than experienced it as a victim). The perpetration measures were more internally consistent than the victimization measures (biased perpetration alpha = .78, nonbiased perpetration alpha = .86). As with victimization, bullying perpetration was dichotomized such that 1 = at least once (in the past few months). Youth were categorized as either nonperpetrators (i.e., did not perpetrate any type of bullying), biased bullying perpetrators (i.e., perpetrated any bullying involving bias), or nonbiased bullying perpetrators (perpetrated only bullying not involving bias).

#### Covariates

In addition to demographic characteristics (see above in “Sample” section), several covariates were included to account for the potential risk factors for biased victimization aside from bullying perpetration. The covariates included a dichotomous indicator of whether the student perceives themselves to be under or overweight (51% of the sample), a dichotomous indicator of low parental monitoring (created from two measures to indicate any student who reported that either their mother/female guardian or their father/male guardian, if they have one, does not know anything about where they are after school [18%]), and the number of nights/evenings per week usually spent with friends, measured continuously (0–6; mean = 2.25). A dislike school measure asked the students how, at present, they feel about school (1 = I like it a lot, 4 = I don’t like it at all; mean = 2.08).

The remaining covariates were coded as ever/never in the specified time period (ever = at least once). These variables were been in a fight (in the past 12 months; at least once = 33%), been drunk (in the past 30 days; at least once = 15%), carried a weapon (including a gun, knife, or club, in the past 30 days; at least once = 15%), and the respondent’s perception of how many of their friends drink alcohol (1 = a few friends or more; 54%).

### Analytic Strategy

Prior to the hypothesis tests, descriptive statistics by type of bullying victimization were presented to show how the profile of youth who experienced biased, nonbiased, and no bullying victimization differed (Table 2).

**Table 2.** Descriptive Statistics by Type of Bullying Victimization, HBSC 2009–2010 (*N* = 8,739).

	No Victimization	Any Biased	Nonbiased Only
Variable	Percent/Mean (SE)	Percent/Mean (SE)	Percent/Mean (SE)
Bullying perpetration			
No perpetration	75	34	57
Any biased	7	31	7
Nonbiased only	18	35	36
Covariates			
Male	59	44	48
Female	41	56	52
Age: 10–13	37	40	45
Age: 14–18	63	60	55
Non-Hispanic White	52	54	56
Non-Hispanic Black	16	14	14
Hispanic	15	13	14
Two or more ethnicities	6	9	7
Asian	4	4	3
Another race/ethnicity	7	6	5
Not born in the United States	8	10	7
Go to bed/school hungry	19	35	26
Urban	26	23	26
Suburban	44	44	42
Rural/other	30	33	33
I am under or over weight	45	60	51
Low parental monitoring	15	24	19
Nights with friends	2.33 (0.06)	2.29 (0.06)	2.05 (0.07)
Been in a fight	28	44	33
Been drunk	12	22	12
Carried a weapon	12	22	12
Any friends drink alcohol	51	63	51
Do not like school	2.02 (0.02)	2.19 (0.03)	2.06 (0.02)

*Note.* Estimates weighted to adjust for the complex survey design; 20 imputed datasets. HBSC = Health Behavior in School-aged Children; SE = standard error.

#### A Biased Bullying Victim-Offender Overlap

To address research question 1, predictive modeling in the form of multinomial logistic regression was employed to predict the victimization types (biased, nonbiased, none) by the perpetration types (biased, nonbiased, none; Table 3) accounting for all covariates. Multinomial logistic regression was used because the outcome was nominal (type of bullying victimization). Whether two associations significantly differed from each other was determined by examining whether the confidence intervals for the two estimates of interest overlapped. This analytic strategy was used because it enabled assessment of whether there was an association between biased victimization and biased perpetration (i.e., a victim-perpetrator overlap) that was greater than the associations between biased and nonbiased victimization and perpetration (indicating type specificity).

**Table 3.** RRRs and 95% CI for Multinomial Logistic Regression Predicting Bullying Victimization (*N* = 8,739).

	Biased (vs. No)		Nonbiased (vs. No)		Biased (vs. Nonbiased)	
Variable	RRR	95% CI	RRR	95% CI	RRR	95% CI
Bullying perpetration (ref = no perpetration)						
Biased	11.33***	[8.88, 14.47]	1.67***	[1.26, 2.21]	6.80***	[5.18, 8.93]
Nonbiased	4.28***	[3.55, 5.17]	2.71***	[2.26, 3.25]	1.58***	[1.29, 1.93]
Covariates						
Female	2.76***	[2.30, 3.30]	1.76***	[1.55, 2.01]	1.57***	[1.32, 1.86]
Age 14–18 (ref = 10–13)	0.88	[0.62, 1.25]	0.96	[0.70, 1.30]	0.92	[0.67, 1.27]
Black (ref = White)	0.77	[0.52, 1.15]	0.92	[0.67, 1.27]	0.84	[0.59, 1.21]
Hispanic	0.66*	[0.44, 0.99]	0.77	[0.57, 1.05]	0.85	[0.61, 1.19]
Two+race/ethnicities	1.26	[0.85, 1.85]	0.99	[0.66, 1.46]	1.28	[0.95, 1.72]
Asian	0.92	[0.63, 1.35]	0.58**	[0.40, 0.84]	1.58	[0.98, 2.53]
Another race/ethnicity	0.74	[0.49, 1.12]	0.69	[0.45, 1.05]	1.07	[0.75, 1.54]
Not born in the United States	1.66**	[1.18, 2.33]	1.12	[0.86, 1.46]	1.48*	[1.10, 2.00]
Go to bed/school hungry	1.86***	[1.49, 2.33]	1.36**	[1.10, 1.69]	1.36**	[1.14, 1.63]
Urban (ref = Suburban)	0.80**	[0.68, 0.93]	3.11***	[2.69, 3.59]	0.26***	[0.22, 0.30]
Rural/other	1.16***	[1.08, 1.24]	1.20***	[1.13, 1.29]	0.96	[0.89, 1.03]
I am under/over weight	1.59***	[1.36, 1.86]	1.23**	[1.06, 1.43]	1.29**	[1.09, 1.52]
Low parental monitoring	1.17	[0.93, 1.47]	1.16	[0.98, 1.38]	1.01	[0.82, 1.24]
Nights with friends	0.93**	[0.89, 0.98]	0.92***	[0.89, 0.96]	1.01	[0.96, 1.06]
Been in a fight	1.69***	[1.34, 2.13]	1.36**	[1.10, 1.68]	1.24*	[1.01, 1.53]
Been drunk	1.12	[0.89, 1.42]	0.91	[0.74, 1.13]	1.23	[0.97, 1.55]
Carried a weapon	1.32*	[1.04, 1.67]	1.03	[0.74, 1.43]	1.28	[0.95, 1.73]
Friends drink alcohol	1.11	[0.87, 1.42]	0.99	[0.83, 1.18]	1.12	[0.91, 1.38]
Do not like school	1.05	[0.94, 1.17]	1.02	[0.93, 1.11]	1.03	[0.93, 1.14]

*Note.* In an alternative model specification with the bullying perpetration reference category set to nonbiased perpetration, the RRR for biased perpetration was 2.65 (95% CI [2.09, 3.34]) for the biased (vs. no) victimization outcome, 0.62 (95% CI [0.47, 0.81]) for the nonbiased (vs. no) victimization outcome, and 4.31 (95% CI [3.29, 5.65]) for the biased (vs. nonbiased) victimization outcome. All models control for school fixed effects (estimates not shown). RRR = relative risk ratio; 95% CI = confidence interval at the 95th percent level; NH = non-Hispanic.

* *p* < .05. **p < .01. ***p < .001.

Since the data analyzed herein are cross-sectional, it was not possible to determine temporal ordering (i.e., whether the victimization occurred before or after the perpetration). Victimization was treated as the dependent variable for the predictive analyses (rather than perpetration) because most existing literature on biased bullying examines factors associated with victimization (e.g., Galán et al., 2021). By treating biased victimization as the dependent variable, this study was able to demonstrate the influence of biased perpetration net of other known risk factors for biased victimization. However, supplemental analyses present the results with perpetration as the dependent variable (see Appendix Table A2).

To test hypotheses 1 and 2, a multinomial logistic regression model predicting biased and nonbiased victimization in comparison to no victimization was estimated. Type of bullying perpetration was the focal independent variable, and all covariates were included. To test hypothesis 3, the same model was re-estimated with the only difference being that the outcome reference category was changed to nonbiased victimization.

#### Prevalence of the Biased Bullying Victim-Offender Overlap by Student Demographics

To address research question 2 and test hypothesis 4, predicted probabilities of experiencing no victimization, biased victimization, and nonbiased victimization by the perpetration types were calculated (derived from the models in Table 3). The probabilities were estimated for the full sample as well as for different demographic profiles of students, as guided by hypothesis 4 and by the first component of the analysis (e.g., see Long & Freese, 2014; Table 4). Predicted probabilities were calculated to assess which biased bullying perpetrators had the highest risk of biased bullying victimization after controlling for confounding influences. Predicted probabilities were used because they present results in terms of the natural measured metric of the dependent variable (i.e., % of students).

**Table 4.** Predicted Probabilities of Bullying Victimization by Perpetration for Full Sample and Among Different Profiles of Students (*N* = 8,739).

Bullying Perpetration	No victimization	Biased	Nonbiased	%
Panel A: Full sample				
No perpetration	64.14	13.68	22.18	100
Biased	25.04	60.52	14.43	100
Nonbiased	35.06	32.03	32.91	100
Panel B: Males				
No perpetration	71.64	9.44	18.93	100
Biased	34.09	50.89	15.02	100
Nonbiased	43.83	24.73	31.44	100
Panel C: Females				
No perpetration	54.69	19.86	25.45	100
Biased	16.97	69.86	13.17	100
Nonbiased	26.19	40.73	33.08	100
Panel D: Not born in the United States				
No perpetration	58.03	19.69	22.28	100
Biased	18.23	70.10	11.67	100
Nonbiased	28.61	41.57	29.82	100
Panel E: Go to school/bed hungry				
No perpetration	56.28	19.14	24.58	100
Biased	17.91	69.04	13.04	100
Nonbiased	27.46	40.00	32.54	100
Panel F: Youth who report being under/over weight				
No perpetration	60.55	16.23	23.22	100
Biased	21.38	64.96	13.66	100
Nonbiased	31.36	36.01	32.63	100
Panel G: Females not born in the United States				
No perpetration	47.75	27.58	24.67	100
Biased	11.90	77.86	10.25	100
Nonbiased	20.51	50.73	28.76	100
Panel H: Females who go to bed/school hungry				
No perpetration	46.15	26.72	27.13	100
Biased	11.71	76.82	11.47	100
Nonbiased	19.70	48.86	31.43	100
Panel I: Females who report being under/over weight				
No perpetration	50.69	23.15	26.16	100
Biased	14.21	73.56	12.23	100
Nonbiased	22.95	44.89	32.16	100

*Note.* Predicted probabilities were derived from the multinomial logistic regression models accounting for all covariates listed in Table 3 (covariates held at the mean). Demographic profiles were chosen as guided by hypothesis 4 and the findings from the first component of the analysis.

Data were tested for normality prior to the statistical tests. The clustering of students within schools was accounted for using school fixed effects. All analyses were adjusted for imputation and the complex survey design using the MI and SVY commands in STATA 17.

## Results

### Descriptive Results

Biased bullying victimization was experienced by 27% of the sample (Table 1). Nonbiased bullying victimization (only) was experienced by 24% of the sample. Forty-nine percent did not experience bullying victimization. Relative to biased bullying victimization, a smaller percentage of students perpetrated biased bullying (13%). Perpetrating nonbiased bullying (only) was reported by 27% of students. For both biased victimization and biased perpetration, sexually biased bullying was the most prevalent bias type (see Appendix Table A1).

About 7% of students who experienced no victimization and nonbiased victimization, respectively, perpetrated biased bullying. Thirty-one percent of youth who were biased bullying victims perpetrated biased bullying (see Table 2). Some covariate characteristics (e.g., being under/overweight) contained a higher percentage of biased than nonbiased victims. Other covariates, such as race/ethnicity, were similarly distributed across the victimization groups.

### Research Question 1: A Biased Bullying Victim-Offender Overlap

#### Hypothesis 1: Predicting Biased Victimization Relative to No Victimization

The first column in Table 3 presents the results of the multinomial logistic regression models predicting biased (vs. no) victimization accounting for the covariates. Biased bullying perpetration (vs. no perpetration) was positively associated with biased bullying victimization (vs. no victimization) by a factor of 11.33 (95% CI [8.88, 14.47]). Nonbiased bullying perpetration (vs. no perpetration) was also positively associated with biased bullying victimization (relative risk ratio = 4.28; 95% CI [3.35, 5.17]). The association between biased perpetration and biased victimization was greater than the association between nonbiased perpetration and biased victimization, as indicated by the nonoverlapping confidence intervals for the two perpetration estimates (first column).

#### Hypothesis 2: Predicting Nonbiased Victimization Relative to No Victimization

The middle column in Table 3 presents the results predicting nonbiased (vs. no) victimization. Biased perpetration (vs. no perpetration) was associated with a 1.67 factor increase in the relative risk of nonbiased (vs. no) victimization (95% CI [1.26, 2.21]). The association between biased perpetration and nonbiased victimization was lesser than the association between biased perpetration and biased victimization (see Table 3).

#### Hypothesis 3: Predicting Biased Victimization Relative to Nonbiased Victimization

The third column in Table 3 presents the results predicting risk of biased (vs. nonbiased) victimization. Biased perpetration (vs. no perpetration) was positively associated with biased (vs. nonbiased) victimization by a factor of 6.80 (95% CI [5.18, 8.93]). Nonbiased perpetration (vs. no perpetration) was positively associated with biased (vs. nonbiased) victimization by a factor of 1.58 (95% CI [1.29, 1.93]). The association between biased perpetration and biased victimization was greater than the association between nonbiased perpetration and biased victimization. Being female, not born in the United States, going to school/bed hungry, perceiving oneself as under or over weight, and been in a fight were all positively associated with biased (vs. nonbiased) victimization as well.

### Research Question 2: Prevalence of the Overlap by Student Demographics

#### Hypothesis 4: Predicted Probabilities of Biased Victimization among Biased Perpetrators

Table 4 presents the predicted probabilities of victimization type by perpetration type. In the full sample (Panel A), 14% of youth who had not perpetrated any bullying were biased bullying victims. Thirty-two percent of youth who perpetrated nonbiased bullying were biased victims, and 61% of youth who perpetrated biased bullying were biased victims. Fifty-one percent of males who perpetrated biased bullying were victimized by biased bullying, whereas 70% of females who perpetrated biased bullying were victimized by biased bullying (Panels B and C). Overall, the predicted probabilities of biased victimization among biased perpetrators were highest for females who go to school/bed hungry (77%) and who were not born in the United States (78%).

### Supplementary Analyses

Supplementary models assessed whether and how the victimization types were statistically predictive of the perpetration types (rather than the reverse; see Appendix Table A2). The results were overall similar to the main results, such that there was a strong positive association between biased victimization and biased perpetration. However, nonbiased victimization was associated with *reduced* risk of biased (vs. nonbiased) perpetration (see column 3 in Appendix Table A2). This finding suggests that although *any* form of bullying perpetration is associated with increased risk of experiencing biased victimization, any form of bullying victimization is not likewise associated with increased risk of biased bullying perpetration.

## Discussion

The school bullying literature has established that some adolescents are both victims and perpetrators (Menesini & Salmivalli, 2017). Little research has examined whether students bully others in ways that mirror victimization experiences (see Galán et al., 2021; Hatchel et al., 2020 for exceptions), and almost no work has focused specifically on whether students who perpetrate biased bullying are also victimized by biased bullying. Several findings from this analysis of the biased bullying victim-offender overlap emerged, which contribute to understanding of prejudicial bullying behavior in the school setting.

### Research Question 1: A Biased Bullying Victim-Offender Overlap

Based on social learning (Sutherland, 1947), situational peer influence (Weerman et al., 2018), and cultural theories (Berg & Schreck, 2022), this study tested for a biased bullying victim-offender overlap among adolescents. It was hypothesized that biased perpetration would be positively associated with biased victimization (relative to both nonbiased victimization and no victimization), and that this association would be greater than those between biased and nonbiased perpetration and victimization.

The results supported these expectations (and hypotheses 1, 2, and 3). Youth who perpetrated biased bullying had a higher risk of biased than nonbiased victimization (and no victimization). The associations between biased perpetration and biased victimization were greater than the associations between biased and nonbiased victimization and perpetration. Thus, youth who bully others are not only at risk of being the victims of bullying generally, but they are specifically more likely to be victims of the type of bullying they perpetrate. These results suggest that the bullying victim-perpetrator overlap is type specific, which contrasts with explanations implying generality, such that perpetration is associated with the elevated risk of any form of victimization indiscriminately (Pridemore & Berg, 2017).

These findings could reflect the social learning of deviance (Sutherland, 1947), either through value transference—in that some youth come to believe that prejudicial bullying is an appropriate way to handle conflict—or a result of behavioral reinforcements within the peer group (Weerman et al., 2018). For example, the observed social rewards that other students receive as a result of perpetrating biased bullying may encourage imitation of the behavior. Levin and McDevitt (1993) posit that the prejudice harbored by thrill-seeking bias offenders is superficial, suggesting that many youths engage in the behavior to adhere to norms rather than out of true animosity. This explanation is consistent with the hate crime and school violence literatures, which suggest that both biased aggression (Levin & Reichelmann, 2015) and general peer victimization (Faris & Felmlee, 2014) are perpetrated to gain status.

In line with cultural explanations of the overlap (Berg & Schreck, 2022), youth might perpetrate biased bullying in schools where social norms imply that biased victimization ought to be reacted to with discriminatory attacks in return or to other students. If the victimization occurred before the perpetration, the biased bullying victim-offender overlap could have been due to direct retaliation (Frey et al., 2015). Students might victimize the *same student* who bullied them in a similar manner for revenge. Future studies should investigate whether this is the case empirically using sociocentric data.

Victims could also have become perpetrators after experiencing distress from the victimization. Galán et al. (2021) suggest that biased victimization may lead to externalizing behaviors such as bullying, remarking how “perceived stigma, minority stress, and systemic disempowerment” factor into peer interactions (Galán et al., 2021, p. 8). However, most accounts of this explanation do not suggest type specificity (Ruback et al., 2014).

Regardless of which of these potential mechanisms underlie the overlap, this study elucidated an important property of biased bullying: it is not unidirectional. Since the victimization and perpetration of biased bullying involves many of the same students, biased bullying likely reflects an interplay between multiple, intersecting identities (privileged and minoritized), which combine relationally in varied ways across situations to influence involvement in biased bullying (Chakraborti & Garland, 2012). These findings challenge narratives of biased aggression, which focus only on particular strands of victims and suggest that applying the target congruence framework may oversimplify risk (Ellonen et al., 2021).

### Research Question 2: Prevalence of the Overlap by Student Demographics

Females, immigrant youth, and youth with family financial difficulties had the highest probabilities of being biased bullying victim-perpetrators. Among females whose families have financial difficulties or who were not born in the United States, more than *three quarters* of biased perpetrators were victimized by biased bullying. The finding for immigrant youth is consistent with hypothesis 4 and studies which find that immigrant background is associated with biased victimization (Maynard et al., 2016) and perpetration (Bayram Özdemir et al., 2020). Though family poverty is associated with the risk of being bullied in general (Tippett & Wolke, 2014), and males perpetrate biased bullying more often than females (Bayram Özdemir et al., 2016), past research has not indicated that students with these characteristics are likely to be involved with prejudicial aggression as *victim-bullies.*

That the biased bullying victim-offender overlap was strongest for females who were not born in the United States and who come from families with financial difficulties may align with explanations for biased aggression that emphasize its use to gain social status (e.g., Levin & Reichelmann, 2015). Due to their socially devalued characteristics, female, poor, and immigrant youth have an increased risk of being victimized by biased bullying (see Table 3), which may lead to them perpetrate biased bullying to gain status and/or to reduce their future victimization risk. This may be especially likely in school contexts or peer environments that provide social rewards for prejudicial bullying.

The finding of high female involvement in the biased bullying victim-offender overlap could also be related to the type of biased bullying that was most common in these data: sexually biased bullying. A qualitative study of biased bullying found that girls’ role in gendered and sexualized bullying was “spotlighted,” or disproportionately noticed and talked about (Mishna et al., 2020). Conversely, boys’ role as perpetrators or victims of gendered and sexualized bullying was largely invisible, in that students (of any gender) described boys as less blameworthy for these incidents. Another potential explanation is that boys are involved in sexual bullying as often as girls but do not interpret or report it as bullying.

### Associations Between the Covariates and Biased Victimization

This analysis also revealed the risk factors of biased victimization after accounting for bullying perpetration. Non-White racial/ethnic groups did not have higher odds than White youth of biased bullying victimization in these data. This finding contrasts with prior research, which finds that non-White youth are disproportionally likely to be victims of biased bullying (Utley et al., 2022). Like the findings for gender, this might be due to the high prevalence of sexually biased bullying.

Being an immigrant, experiencing familial financial hardship, perceiving oneself as under/overweight, and having been in a fight were also positively associated with biased victimization. The findings for immigrant status and perceiving oneself as under/overweight are consistent with the findings of prior studies (Bucchianeri et al., 2013; Maynard et al., 2016). The author is not aware of any previous work finding that being in fights or financial hardship is associated with an increased risk of experiencing biased bullying victimization.

### Implications

The finding of a biased bullying victim-perpetration overlap implies that it may be fruitful for intervention efforts to target group norms pertaining to prejudicial aggression. Rather than focusing on perpetrators or “problematic” students, initiatives could follow a “whole school” approach and acknowledge that bullying is a group phenomenon resulting from processes at various levels (Kärnä et al., 2011). Bystander interventions can be effective for curbing school bullying (Salmivalli et al., 2011), and youth tend to overestimate the extent to which their peers approve of bullying (Dillon & Lochman, 2022). Programs focusing on changing the school climate around prejudicial bullying could therefore prove efficacious for reducing the behavior (Waasdorp et al., 2011). Indeed, researchers emphasize that the classroom reward structure surrounding bullying is crucial: *“if fewer children rewarded and reinforced the bully, and if the group refused to assign high status for those who bully, an important reward for bullying others would be lost”* (Salmivalli, 2010, p. 114).

Most of the existing programs that combat bullying do not focus specifically on prejudice (Earnshaw et al., 2018), even though specific focus is needed to break the cycle of bias. Encouraging positive ways to achieve resect among peers (e.g., by emphasizing academic success) could help combat retaliatory norms (SooHyun et al., 2024). As many students involved in biased bullying in this study were also involved in nonbiased bullying, existing anti-bullying programs can be tailored to reach biased bullying perpetrators as well.

### Limitations

The data analyzed in this study are cross-sectional, so drawing conclusions about causal explanations for the biased bullying victim-offender overlap is not appropriate. Future research should utilize longitudinal data and examine whether biased perpetrators are likely to become future victims, or whether biased victims are likely to become perpetrators at a later date. This study was also not able to test whether the bullying was perpetrated in retaliation (which would require sociocentric data). Since these data are not sociocentric, it was also not possible to examine whether bystanders perceived these incidents as biased.

The HBSC survey did not ask students about all types of biased bullying. For example, biased bullying related to (dis)ability status was not captured in these data. Sexually biased bullying was measured broadly in the HBSC survey and might have encompassed both gender and sexual orientation biases. This study also was not able to assess biased bullying involving multiple biases. It could be, for example, that victims who experienced more than one type of biased victimization (e.g., gender and racial bias) were more likely to become biased perpetrators than victims who experienced one type. These analyses also did not examine the overlap for students who hold multiple minoritized identities.

Some types of biased victimization could have been underreported due to the stigma associated with victimization and discrimination. Biased perpetration may also have been underreported by students who feel shame about it. The goal of this study was to test for the presence of a type-specific overlap for biased bullying in a nationally representative sample of students, and it was not possible to directly test the theoretical mechanisms discussed.

## Conclusion

This study provided a unique contribution to the literature on adolescent bullying and prejudicial aggression by examining whether there is evidence of a type-specific victim-offender overlap for biased bullying in a national sample of American youth. Results revealed that perpetrators of biased bullying were more likely to be victims of biased bullying than nonbiased bullying. The biased bullying victim-perpetrator overlap was most prevalent among females, immigrant youth, and youth whose families experience financial hardship. These results have important implications for advancing scholarly understanding of early manifestations of prejudicial attacks and spotlight one avenue by which educational institutions may magnify existing social inequalities.

## Appendix

**Table A1.** Prevalence of Bullying Victimization and Perpetration Bias Types, HBSC 2009/2010 (*N* = 8,739).

Victimization Bias Types (If Any Biased Victimization; Not Mutually Exclusive)	
Sexually biased	81%
Racially biased	41%
Religion-based bias	28%
Perpetration Bias Types (If Any Biased Perpetration; Not Mutually Exclusive)	
Sexually biased	76%
Racially biased	53%
Religion-based bias	39%

*Note.* Racially biased bullying included incidents perceived to be motivated by race or color, sexually biased included incidents involving sexual jokes, comments, or gestures, and religion-based bias involved any incidents related to religion. HBSC = Health Behavior in School-aged Children.

**Table A2.** RRR and 95% CI for Multinomial Logistic Regressions Predicting Bullying Perpetration (*N* = 8,739).

	Biased (vs. No)		Nonbiased (vs. No)		Biased (vs. Nonbiased)	
Variable	RRR	95% CI	RRR	95% CI	RRR	95% CI
Bullying victimization (ref = no victimization)						
Biased	12.29***	[9.64, 15.67]	4.23***	[3.51, 5.10]	2.90***	[2.29, 3.69]
Nonbiased	1.75***	[1.33, 2.31]	2.70***	[2.25, 3.24]	0.65**	[0.49, 0.86]
Covariates						
Female	0.42***	[0.33, 0.53]	0.97	[0.82, 1.16]	0.43***	[0.33, 0.56]
Age 14–18 (ref = 10–13)	0.96	[0.71, 1.32]	1.03	[0.81, 1.32]	0.93	[0.67, 1.31]
NH Black (ref = NH White)	2.07***	[1.38, 3.11]	1.16	[0.91, 1.49]	1.78**	[1.20, 264]
Hispanic	1.54*	[1.06, 2.24]	1.08	[0.86, 1.34]	1.43*	[1.04, 1.98]
Two+race/ethnicities	0.95	[0.60, 1.49]	0.86	[0.66, 1.11]	1.10	[0.74, 1.65]
Asian	1.16	[0.69, 1.96]	0.97	[0.66, 1.42]	1.20	[0.68, 2.10]
Another race/ethnicity	1.06	[0.68, 1.67]	0.96	[0.68, 1.36]	1.10	[0.74, 1.65]
Not born in the United States	0.74	[0.50, 1.08]	0.89	[0.63, 1.24]	0.83	[0.57, 1.21]
Go to bed/school hungry	1.22	[0.97, 1.53]	1.08	[0.89, 1.32]	1.12	[0.87, 1.45]
Urban (ref = Suburban)	0.13***	[0.11, 0.16]	0.76***	[0.67, 0.87]	0.17***	[0.14, 0.21]
Rural/other	0.21***	[0.19, 0.24]	1.03	[0.95, 1.11]	0.21***	[0.18, 0.23]
I am under/over weight	1.14	[0.93, 1.40]	1.04	[0.91, 1.19]	1.09	[0.88, 1.35]
Low parental monitoring	1.49**	[1.15, 1.95]	1.27*	[1.00, 1.60]	1.18	[0.90, 1.54]
Nights with friends	1.09**	[1.03, 1.15]	1.05*	[1.01, 1.08]	1.04	[0.99, 1.09]
Been in a school fight	1.76***	[1.40, 2.23]	1.59***	[1.34, 1.89]	1.11	[0.88, 1.39]
Been drunk	1.78***	[1.36, 2.33]	1.33**	[1.09, 1.62]	1.34	[0.97, 1.84]
Carried a weapon	2.67***	[2.06, 3.46]	1.33*	[1.06, 1.68]	2.00***	[1.47, 2.73]
Friends drink alcohol	1.93***	[1.44, 2.58]	1.92***	[1.67, 2.22]	1.00	[0.77, 1.30]
Do not like school	1.11	[1.00, 1.25]	1.06	[0.97, 1.17]	1.05	[0.95, 1.16]

*Note.* In an alternative model specification with the bullying perpetration reference category set to nonbiased perpetration, the RRR for biased perpetration is 7.01 (95% CI [5.35, 9.18]) for the biased (vs. no) victimization outcome, 1.57 (95% CI [1.28, 1.91]) for the nonbiased (vs. no) victimization outcome, and 4.47 (95% CI [3.42, 5.86]) for the biased (vs. nonbiased) victimization outcome. All models control for school fixed effects (estimates not shown). RRR = relative risk ratio; 95% CI = confidence interval at the 95th percent level.

* *p* < .05. ***p* < .01. ****p* < .001.
